# Industry 4.0 and Precision Livestock Farming (PLF): An up to Date Overview across Animal Productions

**DOI:** 10.3390/s22124319

**Published:** 2022-06-07

**Authors:** Sarah Morrone, Corrado Dimauro, Filippo Gambella, Maria Grazia Cappai

**Affiliations:** 1 Department of Veterinary Medicine, University of Sassari, 07100 Sassari, Italy; s.morrone@studenti.uniss.it; 2 Research Unit of Animal Breeding Sciences, Department of Agriculture, University of Sassari, 07100 Sassari, Italy; dimauro@uniss.it; 3 Research Unit of Agriculture Mechanics, Department of Agriculture, University of Sassari, 07100 Sassari, Italy; gambella@uniss.it

**Keywords:** precision livestock farming (PLF), internet of things (IoT), Industry 4.0, animal production

## Abstract

Precision livestock farming (PLF) has spread to various countries worldwide since its inception in 2003, though it has yet to be widely adopted. Additionally, the advent of Industry 4.0 and the Internet of Things (IoT) have enabled a continued advancement and development of PLF. This modern technological approach to animal farming and production encompasses ethical, economic and logistical aspects. The aim of this review is to provide an overview of PLF and Industry 4.0, to identify current applications of this rather novel approach in different farming systems for food producing animals, and to present up to date knowledge on the subject. Current scientific literature regarding the spread and application of PLF and IoT shows how efficient farm animal management systems are destined to become. Everyday farming practices (feeding and production performance) coupled with continuous and real-time monitoring of animal parameters can have significant impacts on welfare and health assessment, which are current themes of public interest. In the context of feeding a rising global population, the agri-food industry and industry 4.0 technologies may represent key features for successful and sustainable development.

## 1. Introduction

The global human population is estimated to reach 9 billion by 2050 [1]. Following, the Food and Agriculture Organization of the United Nations (FAO) stated that, in order to keep up with the rising human population, global food production would have to increase by 70% [2]. Similarly, the global demand for meat and other animal food products is increasing progressively. Furthermore, as economic conditions in developing countries improve, a shift in food choice towards animal based protein is expected, further increasing the demand [3]. Economies of scale compels farmers to expand and grow their operations, resulting in higher output. Consequently, the occurrence of farms with a larger number of raised heads, monitored by fewer farmers, is expected. Moreover, the average age of farmers is increasing (average of 58 years old in the USA and Europe, 63 in Japan) particularly in industrialized countries. Given these factors (increasing scale of farms and number of animals raised), the observational capacity and hands on experience which farmers relied on in the past is no longer sufficient to ensure efficient daily herd management [4,5].

Besides the increasing demand for animal-derived products, there are two additional critical issues driving the implementation of modern livestock management practices that deserve attention: livestock environmental impact management and animal welfare [6,7,8]. These topics are sensitive issues that are heavily regulated by national and international law. Given the significance of these issues, methods for monitoring and controlling mandatory and voluntary regulatory requirements must be swift, effective and efficient. Hence, the livestock industry is becoming increasingly reliant on automated real-time monitoring. Terms such as precision livestock farming (PLF), precision agriculture (PA), smart farming and industry 4.0 are more and more widespread. To date, various countries and major world organizations make use of these terms as cornerstones for the development of sustainable and user-friendly farm management. 

Given this background, this review aims to describe the state of the art of industry 4.0 in agriculture and precision livestock farming (PLF). In the first part, the core concepts of modern farming systems are reported. In the second part, current knowledge regarding the applications of these technologies in different animal production systems as well as respective production processes are explored. Lastly, the advantages and disadvantages of this new farming system are compared to different extents.

## 2. The Rise of Technology and Gaps in Current Farming Systems: Core Concepts for Automatic, Non-Invasive, Real Time Data Storing and Sharing

### 2.1. Industrial Revolution: From Industry 1.0 to 4.0

The twentieth and twenty-first centuries can be considered as an industrial arena in which significant development has been brought forth [9]. Roughly, four technological revolutions have so far occurred, going from Industry 1.0 to 4.0. Industry 1.0 progressed human activities from slow and small scale agricultural production to an industrialized society (from the eighteenth to nineteenth century, globalization of the market). At this time, there was only one dimension to the industrial goods market: product volume (simple market). The economics of Adam Smith’s Wealth of Nations [10] was a central concept in Industry 1.0, where price was defined as an automated tool for changing the mismatch between supply and demand. The range of products was restricted and most commodities were agricultural products. During industry 2.0 (from the turn of the century through the 1980s), industrial goods increased significantly in both volume and variety. Electricity, electronic and mechanical devices, and motorised vehicles represented significant technical advancements. Frederick Taylor’s theory of “Scientific Management” was a milestone in Industry 2.0 [11]. The market had two dimensions: volume and variety (stable market). Industry 3.0 (from the 1980s to the present) was characterized by technological developments that had significant effects on the electronics industry in particular, such as the transition from analogue to digital. Additionally, most electronic products modified their product design from integral to modular, leading to a drastic reduction in average product life cycles (volatile market). Lastly, following the continuous technological advancements of the last decade, industry 4.0 came to be, assuming a progressively central role in the discourse between entrepreneurs, experts, academics, and lawmakers on the future of industrial production systems. Nowadays, industry 4.0 is widely regarded as a radical shift in global economic and production systems [12].

### 2.2. Industry 4.0

The term Industry 4.0 originated in Germany in 2011 and has since rapidly been translated and adopted throughout the world. Specifically, this new paradigm is focused on factory automation, the incorporation of the internet into industrial processes, and the dissemination of information and communication technology (ICT) in order to create intelligent devices, machines, and systems [13]. Industry 4.0 is the result of the 4th industrial revolution and is described as a process leading to fully automated and interconnected industrial production [14]. According to Rüßmann et al. [15], industry 4.0 is an “initiative with technology innovations such as internet of things (IoT), big data, electric vehicles (EV), 3D printing, cloud computing, artificial intelligence (AI) and cyber-physical systems”. Cyber-physical systems interact and collaborate with each other and humans in real time over the IoT. Moreover, both internal and cross-organizational services are offered and used by value chain participants through the Internet of Services [16]. Cloud computing is a kind of internet-based computing that enables on-demand delivery of shared software and hardware data to computers and other equipment. End users are not required to understand or have direct control over the fundamentals of the “cloud” or to possess professional knowledge of it. All they need to know is what kind of resources are required and how the internet can provide suitable services. The five characteristics of cloud computing are: on-demand service, internet access, polling of resources, rapid elasticity and calculability [17]. All in all, IoT links the internet to certain sensors, runs the programs and provides remote control.

### 2.3. Internet of Things (IoT)

The IoT is a network of interconnected devices that communicate, sense, and interact with internal and external environments via embedded technology [18]. As a key technology in Industry 4.0, IoT is changing several aspects of our day-to-day activities by generating a smart connected environment. Smart homes, industrial internet, and wearable devices are some of the examples of daily use [19]. 

Kevin Ashton, British entrepreneur and founder of various start-ups, conceptualized the IoT. The term was coined in 1999 to define a framework wherein the physical world interacts with computers (through data exchange) and ubiquitous sensors. Almost ten years later, between 2008 and 2009, the number of computers connected to this network surpassed the number of inhabitants of our planet. This event can be considered the true birth of the “Internet of Things”, also known as the “Internet of Everything” (IoE). Not only objects, but also processes, data, people, even animals and atmospheric phenomena are evaluated by this approach, all of which can be considered as a variable [20]. 

IoT based smart farming consists of four major components: physical structure, data acquisition, data processing, and data analytics. In order to prevent any unexpected incidences, the physical structure is the most critical element for precision agriculture. The entire system is constructed in such a way that the sensors, actuators, and devices are managed efficiently. Sensors perform various tasks, such as sensing soil, temperature, weather, light and moisture. Likewise, devices perform several control functions, such as detection of nodes, recognition of devices and naming services, etc. Any system or sensor operated through a microprocessor is capable to perform all these tasks and any remote device or computer connected through the internet performs this control process. 

The acquisition of data can be further divided into two sub-components: the acquisition of IoT data and the regular acquisition of data. In this context, seven protocols exist for the acquisition of IoT data. More protocols may be used for the implementation of smart farming, depending on specific needs and conditions. 

Data processing includes a variety of applications, such as image or video processing, decision support systems and data loading and gathering. Any functionality that may work simultaneously to provide other services may be integrated according to the system requirements. 

In precision farming, monitoring includes three key applications: livestock monitoring, field monitoring and monitoring of greenhouse gasses [21]. The IoT allows farmers to oversee livestock through the use of multiple sensors that monitor multiple animal variables such as temperature, heart rate and digestion. Field monitoring applications are designed to report on different conditions such as soil fertility, temperature, humidity, gas content, air and water pressure, and the presence of disease in crops [22]. The use of IoT sensors and devices has also managed to eliminate the need for manual intervention to some extent, constituting a smart greenhouse design able to monitor and regulate various climatic parameters in accordance with the plants’ needs [23].

### 2.4. Big Data

The enormous amount of information that is produced and gathered daily is processed and analysed with the help of Big Data beyond the capabilities of conventional techniques. Big Data serve as the basis for advanced technological and statistical tools, analysing huge amounts of data and extrapolating information. Big Data enable handling and use of this continuously growing dataset in a rapid and easy way. Big Data applications evaluate and distinguish the important from the less important, thus advancing the ability to reach conclusions and facilitate successful knowledge sharing to achieve business goals [20]. One primary feature of Big Data is that it can be used to extract a huge amount of values, which necessitates the use of complex analytical methods. Processing huge quantities of data can be costly, time-consuming, and frustrating [24]. Data for PLF are generated primarily by wireless sensor networks, weather stations, unmanned aerial vehicles (UAVs), and external services. 

The expansion of the IoT and the IoE has resulted in a significant increase in data volume. Cloud computing and big data offer numerous tools for addressing the issue of processing and storing all requirements and constraints associated with smart farming [23].

### 2.5. 5G

Due to poor internet accessibility in rural areas, mobile network devices have not been sufficiently implemented, and even in areas with high-speed connectivity, failures occur due to massive demand. However, in some developed countries, farms with a large number of IoT devices and machines, all of which require a continuous, stable high-speed internet connection to exchange large amounts of data, have been set up [25]. 

The 5G mobile network, which enables large coverage and high spectrum performance with low energy consumption and low-cost devices, is well positioned to promote smart farming. 5G is the 5th generation mobile network technology. To achieve extremely high network speed and low latency, the 5G mobile network makes use of a high-band spectrum [26]. Because of its greater bandwidth, 5G has the capacity to link billions of devices in addition to high data capacity. The maximum data rate of the 5G network per mobile station is 10 Gbps for uplink and 20 Gbps for downlink [27]. In terms of downloading and uploading rates, 5G can outperform predecessing 4G and 4G LTE standards by up 100 times [28]. 5G applications in agriculture and livestock include UAVs, real-time monitoring, online counselling and predictive maintenance, augmented and virtual reality, AI-powered robots, data analytics, and cloud repositories.

### 2.6. Agriculture 4.0

Agriculture 4.0 is similar to Industry 4.0. Hence, Agriculture 4.0 refers to integrated internal and external networking of farming activities. This implies that, for all farm sectors and processes, information in digital form must exist. Using Internet-based portals, Agriculture 4.0 makes it easy to manage large quantities of data, as well as to network within farms [17]. In parallel, similar changes in the manufacturing world, where this phenomenon is marked as “Industry 4.0”, based on a vision for future production occur. Indeed, Agriculture 4.0 is paving the way for the next agricultural evolution, consisting of unmanned operations and automated systems of decision-making. Smart Agriculture, which is based on cloud computing and IoT, where a central computer manages and controls internet-based machines and workers, enhancing production and life quality through more comprehensive and dynamic ways. This is advantageous for the integration of human society and the physical world, and it is seen to represent the third era of digital industrial development following computers and the internet. 

The main IoT technologies are radio frequency identification technology [29,30], sensor technology, sensor network technology and internet communication, which are all involved in the four IoT industrial chain links, namely identification, sensing, processing and distribution of information [16]. 

To achieve device optimization, Agriculture 4.0 would exploit the smart use of data and communication. A huge amount of data can potentially be collected by digital agriculture, and key factors for the implementation of Agriculture 4.0 are analytical skills that enable the efficient use of this data. Other manufacturing sectors have preceded agriculture, in which the advantages of digital technology have materialized and become a source of improved production efficiency as the omnipresent data are effectively used [31]. The competitiveness of a nation’s agriculture and the ability to maintain vital natural resources in a global economic climate will be closely related to its ability to innovate in these aspects of the production system. The question is not whether digital technology should be embraced by the global agricultural industry, but how this adoption process will take place in an environment that allows farmers to capitalize on potential production gains [32].

### 2.7. Precision Livestock Farming (PLF)

The idea of precision agriculture (PA) was presented for the first time in the early 1990s in the USA. In 1997, the House of Representatives described PA as an “integrated information and production based farming system designed to increase the efficiency, productivity and profitability of long-term, site-specific and entire farm production while minimizing impacts on wildlife and the environment”.

While it has long been assumed that biological processes involving living organisms are too complicated to be monitored and regulated automatically, new emerging technologies are providing opportunities to develop fully automated online monitoring and control tools for many of these processes. Animals, like all living organisms are complex, individually distinct and time-variant, referred to as “Complex, Individual and Time-variant” (CIT) systems [33]. This leads to some benefits, such as increased productivity, but the problems associated with this approach are potentially severe. For example, increasing livestock production density would exacerbate the environmental impacts of farm animals on climate, groundwater and air quality, and could bring about animal welfare problems. Scaling up farms while still maintaining individualized treatments and attention to animals is a core issue. As a result, PLF was conceived as the solution to face these issues [34]. PLF can be described as the technological system for real-time monitoring of farmed animals, aimed at managing the temporal variability of the smallest manageable production unit, known as the ‘per animal approach’ [33]. The individual electronic milk meter for cattle was the first significant implementation of PLF, years before the word was coined in 2004. PLF was described by Wathes et al. [35] as the management of livestock production using process engineering principles and technology. The concept of PLF is to develop a management system based on integrated automated control and monitoring of production/reproduction, animal health, welfare, as well as the environmental impact of livestock farming in real time [36,37]. To obtain such a functional control and monitoring system, three conditions must be fulfilled. The first condition is to constantly monitor animal variables, and for this information to be consistently analysed. The second prerequisite is the availability of a realistic prognosis (expectations) on how animal variables will differ or how the animal will respond to environmental changes. The third condition is that these predictions, together with digital measurements, are incorporated into an analytical algorithm for the automated tracking or management of animals and for the online monitoring of animal health and welfare [33]. Although the overall principle of utilizing technology and automation to increase precision in industrial manufacturing is readily transferrable to PLF, the shift from inert products to live animals introduces unique issues that must be considered when building PLF procedures [38]. 

Nowadays, accurate, powerful and low-cost instruments are available and technological advancement in the last decade. These include cameras, microphones, sensors, wireless networking systems, internet connections and cloud storage. The purpose of these technological instruments is not to substitute but rather to help a farmer who still remains the most important aspect of good animal management [39]. 

The great potential of PLF is focused on early alerts, which offer the farmer the power to act as soon as the first signs of impaired welfare or health emerge [40]. Indeed, accurate prediction models have been developed in the context of PLF that send warning messages to farmers based on information from animal and environmental inputs and can help detect any deviation from the usual pattern. Thanks to the detailed information reported with regard to the status of their livestock, farmers may easily take corrective management measures. Continued technological developments and validation of theorical aspects and prototypes contribute to the creation of PLF diagnostic tools that can detect farm problems without manipulation of animals (contactless and non-invasive data gathering) or inducing stress, providing the ability to recognize a disease outbreak days before any farmer would [41]. In this context, the benefits to farmers include improved decision-making, increased attractiveness for young farmers, and a beneficial effect on resolving the end user’s analytical shortcomings through the conversion of raw data to useful information that is currently only obtainable through expert analysis and interpretation. 

PLF is focused on the link between various scientific disciplines and livestock industry’s stakeholders. The PLF approach involves a high degree of cooperation between many fields of study, including animal scientists, veterinary clinicians, technicians, data scientists, engineers and others [42]. The data science task of translating various types of data from different sensors and sources into applicable information is the biggest barrier PLF field applications face. There are two impediments in this regard: (1) the data science issue associated with the development of data-driven techniques capable of generating predictions depending on a variety of measurements from multiple sources; and (2) the translational issue associated with interpreting data-driven predictions to generate an insightful level of knowledge. 

The scale on which the process is managed is implicit in the PLF definition. Generally speaking, the smaller the operation size in which PLF is used, the higher the cost and a greater return on the capital investment is required to justify the more detailed and more accurate management of the enterprise. 

Finally, digitalization provides the ability to make farming more competitive. The advent of Information and Communication Technology (ICT) in the livestock industry and the increasing usage of the IoT have opened a new age of connectivity in which things, people and animals are part of an exchange of data networks, leading to a new philosophy of agriculture [43]. 

In summary, PLF and Industry 4.0 are not two processes that are chronologically divided nor completely independent. Industrial innovation has enabled researchers to use the most innovative systems to change and improve the approach used to manage livestock farming. On the other hand, the use of these real-time and internet-based monitoring technologies spurs industrial innovation [44].

## 3. Animal Productions and Farming Systems

**Figure 1:**
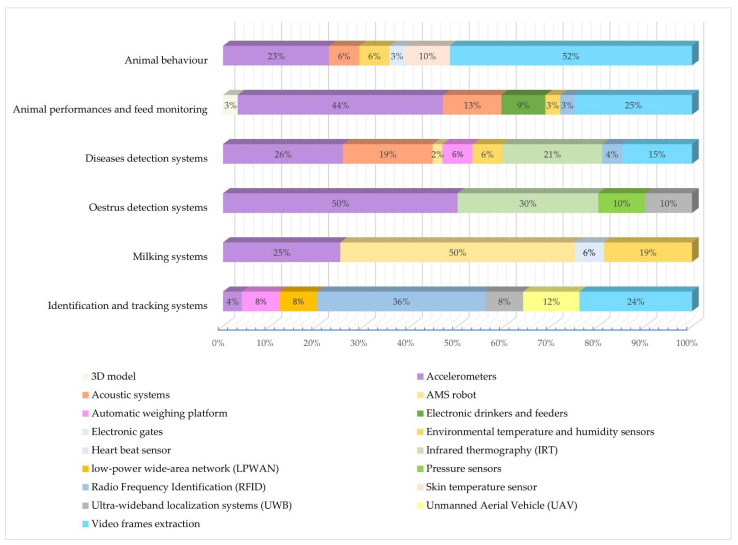
Percentage of the most used sensors per each area of interest in PLF.

**Figure 2:**
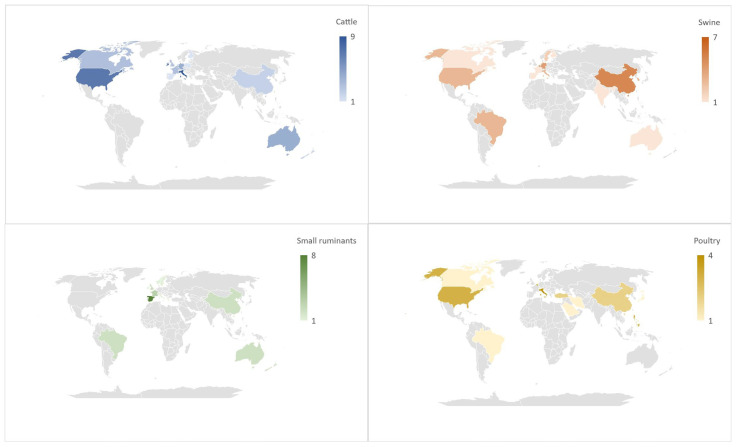
Number of articles related to PLF published worldwide in relation to the species (blue: cattle; orange: swine; green: sheep and goat; yellow: poultry).

A summary of technologies used to monitor parameters in various animal species is listed in Table 1 at the end of this chapter. Furthermore, Figure 1 and Figure 2 schematically summarize the following chapter. The percentage of the different sensors for each area of interest is illustrated (Figure 1), and four maps illustrating the number of articles published in each country for the four animal species considered in this review are depicted (Figure 2).

### 3.1. Cattle

The following section pertains to the applications of PLF in cattle farming. Several of the areas discussed below (identification and tracking systems, illness detection, animal performance, feed monitoring and animal behaviour) are applicable to both beef and dairy cattle and are discussed in detail. For obvious reasons, the automatic milking system (AMS) and oestrus detection are only discussed with regard to dairy cattle.

#### 3.1.1. Identification and Tracking Systems

The use of modern technology in cattle farming began with the automatic identification of animals [45]. Presently, the automation of dairy farms is very much rooted in industrialized countries. Moreover, a framework based on deep learning for identifying beef cattle in feedlots using image sequences was proposed, combining the advantages of Convolutional Neural Network (CNN) and Bidirectional Long Short-Term Memory (BiLSTM) network approaches [46]. Cattle with distinct coat colours (e.g., 560 white, brown, and yellow) as well as cattle with comparable coat colours are effectively identified with a 91% identification accuracy utilizing a 30-frame video. A group of scientists in the United Kingdom conducted research in which they employed a drone-based system in conjunction with an automatic coat pattern recognition system to identify individual cows in freely moving herds [47]. This system, which is both offline and online compatible, reached an extremely high level of accuracy (91.9% and 94.4%, respectively). The use of automatic animal identification and monitoring techniques for extensive farm systems was also investigated, with excellent results [48,49,50,51].

#### 3.1.2. Automatic Milking Systems

One of the earliest PLF developments is the automatic milking system (AMS). All over the world, AMS has revolutionized dairy farming. Besides the milking process is operated by machines, many improvements have been made to how the entire farm system is handled. Milking is no longer carried out in specific sessions; rather, cows freely choose when to be milked in AMS, enabling milking to be spread over a 24 h cycle [52]. A number of studies have demonstrated that storing collected data properly in structured databases is a necessary pre-requisite for developing numerical models capable of characterizing the conditions and performance of individual cows [53] as well as to quantify the effects of specific thermo-hygrometric conditions on dairy production [54,55]. The most recent study, which defined, trained, and tested a model developed using machine learning techniques and the Random Forest algorithm to assess a single cow’s daily milk production in relation to environmental conditions, achieved excellent results, with an overall average error in predicting total milk production of just 2% [56].

#### 3.1.3. Oestrus Detection

The dairy cattle industry subsequently focused on the most common management practices requiring detailed data in order to be optimally approached: feed management, prompt diagnosis of oestrus, and identification of potential disease signs. To date, there are a multitude of systems, sensors and software helping farmers in the management of these activities. Regarding oestrus detection, automated activity systems are varied in their output or variables to be analysed (e.g., step counts, movement acceleration, rumination frequency, lying duration). For example, a neck-mounted device detected 71% of the preovulatory phases, missing solely 13% of the recorded ovulations [57]. Using the same sensors, Aungier et al. [58] reported that 72% of the preovulatory follicular phases were detected accurately, while 32% were false positives. In a following study, an automated activity monitor detected 83% of cows in oestrus [59]. Pedometer measurements detected 51 to 87% of all oestrus periods, according to previous research from the Netherlands [60]. Data on the movement and speed of cows have successfully been exploited for the performance of specific analyses, such as oestrus detection, through an ultra-wide band real-time location system (UWB RTLS) in a free-stall stable [61]. This program was created with the functions necessary for integration into the IoT ecosystem. Along with identifying oestrus, the PLF approach has been used to monitor potential oestrus influencing factors. A Canadian study monitored several temperature and wellness parameters and successfully detected oestrus in cattle using video and infrared cameras [62].

#### 3.1.4. Diseases Detection

Both in dairy and beef cattle, pedometers, along with several other systems, are used in the detection of lameness. Direct and indirect methods have been used individually [63,64,65,66,67] or in combination. In recent years, the complexity of diagnostic systems has increased considerably. Seven variables (walking speed, beats per minute (BPM), daytime activity, milk yield, lactation stage, milk peak flow rate, and milk peak conductivity) have been used to diagnose lameness, reporting a sensitivity of 68.5% and specificity of 87.6% [68]. More recently, sensors capable of evaluating a total of 50 variables have been used to constitute machine learning for the purpose of lameness diagnosis in 229 cattle, showing high precision [69]. Finally, it was described as an end-to-end IoT application that uses threshold-based clustering and machine learning classification to identify lameness in dairy cattle [70,71]. The finding was 87% accurate, indicating an early stage of diagnosis, approximately three days before the appearance of visible or clinical signs of lameness. Recently, studies have evaluated the implementation of a PLF approach for the detection of other diseases commonly present on farms. To this extent, PLF technology may be effective in detecting calving illnesses [72,73,74,75,76] (such as mastitis and ketosis) affecting the mammary glands of dairy cows [76,77,78].

The management of calves is a major challenge in breeding. Among the most common diseases are respiratory disorders. Coughing was monitored in a calf house as a means of detecting cases of respiratory infection before they became too severe [79]. The algorithm developed worked with a precision higher than 80%. More recently, the use of infrared thermography (IRT) was used to measure non-invasively the respiration rate in calves, based on thermal fluctuations around the nostrils during inhalation and exhalation with a high correlation [80]. 

#### 3.1.5. Animal Performances and Feed Monitoring

Observation of dairy cows’ ingestive and ruminal behaviour aids in identification of disease as well as animal productivity, individual grazing intake and animal welfare evaluation, within precision dairy production systems [81]. The Rumi Watch is a jaw movement sensor that can track and differentiate between prehension bites, eating chews and rumination chews. Results of various studies conducted between 2016 and 2021 demonstrate the RumiWatch sensor performed well in counting prehension bites with a concordance correlation coefficient > 0.96, eating chews > 0.95 and rumination chews > 0.96 [82,83,84,85,86,87,88]. 

In the beef industry, the usage of electronic monitoring systems has mainly been used in research settings, e.g., for analysing the effects of feed intake on growth efficiency [89], intake of salt-limited supplements [90], and rumination monitoring [91]. These technologies could easily be modified for implementation in beef cattle production systems to monitor activity, feeding, and drinking behaviour, as well as to serve as tools for inventory management in intensive or extensive production systems. A recent device (BEHARUM), equipped with a three-axial accelerometer sensor, couples the use of Micro Electro-Mechanical Systems and ICT. The sensor, inserted in the halters of Sardinian cow, measures feeding behaviour by detecting accelerations resulting from jaw and head movements [92]. The results obtained showed a grazing, ruminating and resting accuracy of 90.3%, 91.2% and 95.1%, respectively, proving that the BEHARUM device to be a useful tool for automated behavioural monitoring of cows on pasture, as previously described in sheep [93]. 

For beef cattle, the conventional 2D scoring system, focused on expert visual assessment, was converted into a 3D model based on four main classes after a SWOT analysis was performed to determine the strengths, limitations, openings, and challenges of traditional compared to innovative morphology evaluation processes. This new approach was considered to be a good representation of live animal morphology as a non-significant difference (*p* > 0.05) was found between the means of the somatic measures calculated on the virtual 3D model and the real Charolaise bull [94]. 

#### 3.1.6. Animal Behaviour

Animal welfare is a multidisciplinary field of study that encompasses scientific, ethical, economic, cultural, social, religious, and political dimensions. This field is growing rapidly in our society and is one of the primary objective of the OIE [95]. Application of numerous technologies has been researched related to this subject in order to generate effective, efficient, and animal-based indices of wellbeing [96]. Several of the devices covered in the previous section have already been addressed in detail (feeding and drinking behaviour, diseases, and oestrus detection). All of these elements must be taken into account when determining the welfare of animals. As a result, several studies have cross-referenced feeding and lying behaviour data, employing multiple sensors per animal and producing reliable indicators of animal welfare in adult cattle [97] and calves [98,99]. A recent study developed a deep learning framework intelligently integrated with C3D and ConvLSTM networks to monitor and identify dairy animal behaviour like feeding, exploring, grooming, walking and standing. Automated behaviour classification using spatiotemporal characteristics such as C3D and ConvLSTM can significantly increase the accuracy of video-based behaviour categorization [100]. Additionally, IoT-based monitoring systems were assessed in a real-world scenario to see whether they could be used to ease the work of stakeholders (veterinarians, farmers, etc.), while maintaining a high level of efficacy and efficiency. For this purpose, a number of applications capable of effectively merging data from several sensors, generating an accurate representation of an individual animal’s welfare, or welfare on the herd level, have been built [101,102]. 

### 3.2. Small Ruminants

According to a study carried out in 2014, the world’s cattle population was estimated at 1.43 billion while the population of sheep and goat was set at 1.87 billion [103]. Despite this high number of small ruminants, research regarding PLF approaches in sheep and goat are significantly less numerous as compared to cattle. 

The following section highlights the most recent studies on the use of technology and IoT in this sector.

#### 3.2.1. Animal Identification and Automatic Tracking

Since the announcement of the European Union’s legislation on the mandatory identification (ID) and registration of goat and sheep born in 2010 or later, electronic identification of goats has received considerable attention [104]. Following, electronic identification sensors for small ruminants can be found in ear tags [105], ruminal boluses [106], or as injected chips under the skin [106], depending on the application. 

Along with identification, the opportunity for continuously monitoring of herds, especially on grazing farms, is of particular interest. As a result, several recent studies have investigated the use of GPS sensors [107,108] and drones [109,110] to monitor animals in real time, revealing an accuracy of 96–97%.

#### 3.2.2. Automatic Milking Systems

In sheep and goat farms, automation is not widespread. However, in recent years, the demand for automatic milking systems has increased significantly [111]. Due to the unique morphological and anatomical characteristics of each species, machines with specific attributes in terms of the amount of time and negative pressure are required. Various studies have been conducted to perfect these automated milking machines for sheep and goat, with excellent results [112,113,114,115].

#### 3.2.3. Diseases and Oestrus Detection 

In small ruminant farming, reproduction and early illness detection are unquestionably core elements. For this reason, these factors represent areas of interest for automated monitoring. A ram-mounted automatic detector capable of recognizing the ID of ewes and determining male acceptance has been successfully applied to diagnose oestrus (100% oestrus diagnosis) [116]. The same device is also capable of providing quantitative information on both ewe and ram sexual behaviour [117]. Other sensors, such as accelerometers [118] and thermographic cameras [119,120], have been used to track the reproductive characteristics of rams as well as ewes.

Infectious diseases and lameness are extremely common in small ruminant farming. The use of technology for early detection of such conditions has been researched in the context of minimizing economic losses on farms. Body temperature can be an excellent predictor of animal health. In this regard, farmers and veterinarians can employ temperature sensors implanted directly on the animals or external, non-invasive sensors to screen the herd and detect any abnormalities [121,122,123]. Another example is the use of infrared thermography to diagnose lameness in sheep. Recent research showed this technique to have a diagnostic sensitivity of 77% and a specificity of 78% [124,125].

#### 3.2.4. Animal Performances, Feed Monitoring and Animal Behaviour

Grazing and rumination are the most essential activities for ruminants, as most of the day is dedicated to these activities. Continuous monitoring of eating behaviour is critical for ruminant health, productivity and welfare. The use of sensors for the automated collection if data, and software to classify and recognize activities enables substantial progress in overcoming this otherwise challenging tasks [126]. Different types of accelerometers, placed on the ears, neck or legs of the animals, have previously been employed to monitor behaviour, with high sensitivity (99, 93, and 100%, respectively) [127,128,129].

All sensors described in this and the preceding sections can be used to monitor sheep and goat welfare. Given that welfare is impacted by a multitude of circumstances, one of the goals of the PLF technique is to analyse and summarize huge amounts of data in order to provide technical expertise with a specific solution. This is why specific technology has been developed that combines minimally or non-invasive temperature sensors inside and outside the animal, as well as accelerometers and sound detectors [130], to create algorithms and machine learning capable of detecting animal welfare on farms [131] and during transport [132].

### 3.3. Swine

The concept of PLF is not new in the European pig industry, and its use is currently growing rapidly. The first PLF-related study carried out on pigs described how vast amounts of information can be generated by continuous monitoring of pig behaviour [133]. In this way, it is possible to examine animal activity, social interactions, and the health status of animals in such a way as to encourage farmers to enhance their management. According to an industry survey, vision-based monitoring is more widespread in pig farming than in cattle farming [134].

#### 3.3.1. Animal Identification and Automatic Tracking

The first step in improving the management of swine farms is through the automatic identification of individual animals. RFID (radio-frequency identification) transponders have commonly been used to replace ear tags for the purpose of automated tracking. The issue of the RFID transponders was only 88.6% [135].

The RFID system, in combination with molecular analysis (DNA) for traceability, has been implemented to enhance the production of “Suinetto di Sardegna” specifically. Thanks to this study, the cost/benefit ratio of this innovative systems has been evaluated. The results are encouraging although further studies are needed to better define the economic sustainability of this system [136]. 

Computer vision and artificial-intelligent-based methods are beginning to attract interests as an alternative approach to automatically evaluate pigs posture [137], perform animal counts [138], monitor outdoor animals [139], detect the onset of farrowing sows [140] and identify swine’s in camera images [141]. A recent research, published in 2020, used an IoT-based approach for pig facial recognition through a Convolutional Neural Network, resulting an accuracy of 83% [142]. 

#### 3.3.2. Diseases Detection

One of the first studies focusing on real-time monitoring in pig farms used a device capable of recording, storing and elaborating vocalizations. Test animals were subjected to citric acid nebulization in a laboratory setting, resulting in coughing. Recorded sounds emitted by the animals were divided according to a two-cluster classification; ‘cough’ or ‘no cough’, by applying 2-means fuzzy clustering on the spectral distances of nine randomly chosen frequency ranges and integrating the classification result in a sum-neuron. As a consequence, coughing was correctly identified in 92% of the cases [143]. In the following years, numerous studies utilizing cough recognition systems have been conducted [144,145,146,147,148] and such systems are now commercially available (Respiratory Disease Monitor). 

A more recent study employed deep learning, a state-of-the-art method that used the fine-tuned Alex Net model, to make classifications. The results of this study revealed the proposed algorithm to significantly outperform other algorithms. Cough and overall recognition accuracy reached 96.8% and 95.4%, respectively [147]. 

#### 3.3.3. Animal Performances and Feed Monitoring

In addition to monitoring the physical health of animals, the PLF approach can be useful in evaluating animal nutrition. Feeding and drinking behaviour are essential for the optimal growth of pigs in increasingly specialized farms. Both pigs raised for charcuterie and pigs raised for fresh meat production must reach optimal weights in the shortest time possible in order to allow farmers to survive in an increasingly competitive market. In addition, food quality is one of the novel challenges of the sector, along with animal welfare and environmental impact reduction [149]. 

Monitoring of water consumption of pigs coupled with automated analysis detected behavioural changes in diurnal drinking patterns one day before physical symptoms of diarrhoea occurred in the same animals [150]. Furthermore, automatic detection of variations in feeding behaviour was shown to give an indication on whether animals were healthy or not [151,152]. 

In association with feed intake, it is important to control and predict the fattening stage of animals. Parsons describes the development and testing of the first prototype real-time system for the integrated control of pig growth using image analysis [4]. Using artificial neural networks, a vision-based machine has been developed capable of working with multiple physical features for live weight calculation [153]. In this study, weight monitoring, with a relative error of around 3%, was achieved as pigs walked freely under a camera (without having to restrain the animals in any way). Hence, recent technological advances have enabled easy monitoring and estimation of pig fattening from images [154,155].

Because of the development of increasingly precise predictive systems, low-cost depth sensors in associated with linear equations have been used to extract mass data from depth images [156,157]. Moreover, these models have been connected to a system able to produce daily notifications for individual fattening pigs with a sensitivity of 58.0%, specificity of 98.7%, accuracy of 96.7% and precision of 71.1% (in addition to alerting the farmer to the physical state of individual animals) [158]. A study based on an IoT system demonstrated monitoring of daily weight gain and established an accurate relationship between environmental information and pigs’ weight [159]. 

#### 3.3.4. Animal Behaviour

The third aspect of swine farming that can be managed by the PLF approach is welfare. The growing public interest and knowledge regarding animal well-being have led national and international institutions to enact laws and directives to protect the welfare of farm animals. Many factors impact animal welfare and can be used as metrics for automated monitoring. 

The intensive farming conditions of pigs can easily become a cause of stress, especially if animals are ill adapted to a specific environment. One parameter responsible for particular discomfort in this animal species is air temperature and quality (ammonia). For this reason, infrared techniques have been developed for automatic and real-time monitoring of changes in skin temperature, not only to control the physical health status of the animals, but also as an indicator for stress [160,161,162,163,164,165,166,167,168]. In addition to temperature control, stress detection in piglets was achieved with high accuracy (from 65.2% to 93% depending on the parameter sought), owing to software capable of monitoring and evaluating vocalization strength in real time [169]. 

Social interactions between pigs can be a source of stress if stocking density is high. Furthermore, this might lead to conflicts between groups of animals, as well as an impaired end product quality due to physical injuries. Animal welfare can therefore be monitored automatically by screening behavioural incidents of pigs; nose to nose interactions and head to head knocking are early signs of aggressive behaviour [170]. This approach was applied as a convolutional neural network to detect the orientation of pigs and to follow their movements using a Kalman filter algorithm. This system was able to construct a network of social connections and, as a result, evaluate the pigs’ behaviour with a sensitivity of 94.2% and a precision of 95.4% [171]. Other studies recognised aggressive episodes of pigs by processing of picture and video material from pens [172,173]. Although not only visual data is employed, a Kinect Depth Sensor was used to accurately recognize and characterize aggressive behaviour in pigs (detection 95.7% and classification 90.2%) [174].

### 3.4. Poultry

PLF has been adopted in poultry farming systems as well. Indeed, the initial applications of PLF focused mainly on broilers and pigs, and later on cattle. With the introduction of PLF in poultry farming, the large number of animals, intensive production of eggs or meat, and relatively low profit margin could be managed with limited staff. 

#### 3.4.1. Diseases Detection

One of the most common and important health problems on poultry farms is the presence of lameness and leg deformities. Several studies proposed a fully automated monitoring system for early detection of lameness, through the use of cameras [175,176,177,178,179] or wearable sensors, like accelerometers [180]. A real-time automatic system has been used to correlate the presence of leg disorders with environmental conditions [181]. The use of automatic and continuous control systems of the posture of chickens is not used only to recognize lameness: a pilot study used a PLF approach to control air quality in association with the presence of coccidiosis, aiming to distinguish infected from uninfected pens at an early stage (when only 250 oocysts g^−1^ were present in one pen) [182]. Zhuang et al. [176] developed an algorithm for early health status evaluation of chickens with an accuracy of 99.5% through the analysis of images of healthy and sick birds and using a Support Vector Machine model. Apart from that, the use of digital cameras and sound recognition systems could be used to diagnose respiratory diseases, such as infectious bronchitis and Newcastle diseases [183,184]. As described above for pigs and cattle, several studies on precision feeding have also been carried out in chickens. 

#### 3.4.2. Animal Performances and Feed Monitoring

When compared to traditionally fed pullets, precision fed pullets lose less thermal energy, most likely because they are fed continuously, minimizing their need to store and mobilize nutrients [185]. Therefore, it is useful to record and monitor feeding behaviour in poultry farming. 

Feed intake and feeding/drinking behaviour of chickens can be monitored and followed up through the use of different types of sensors. Pecking sounds can be recorded using sound technology and, thanks to the development of different algorithms, these recordings can be converted into real-time analytical evaluations of feed intake and feeding behaviour [186,187,188]. The use of cameras capable of monitoring chickens has also been used to control feeding and drinking behaviour. The eYeNamic system, a three-camera device mounted on the ridge of broiler houses capable of monitoring chicken behaviour 24 h a day, was used to determine the proximity of broilers to pans or drinking lines. Interpretation of the results of this system indicated homogeneity between human monitoring and that of the eYeNamic System [189]. 

The IoT has also been successfully applied in poultry farming; a group of researchers developed an app that sends out notifications whenever water or food containers for hens need to be refilled [190].

#### 3.4.3. Animal Behaviour

Numerous sensors have been used to study animal behaviour and have been shown to be promising tools for determining the welfare condition of broiler breeders in commercial production systems [191]. In restricted and unrestricted environments, tracking systems such as recording devices have been used to classify and distinguish broiler and breeder activity patterns in order to determine their health status [177,192,193,194]. These automated systems have been compared with gold standards in a number of studies. Researchers at Ohio State University conducted a study comparing continuous behavioural observation (the gold standard) to automatic monitoring software and showed that less than 1-min intervals of instantaneous scan sampling can accurately detect high-frequency activities [195]. 

Feeding behaviour is closely related to welfare. In this context, many studies have developed single and integrated systems of acoustic and visual sensors to build algorithms able to evaluate the feeding behaviour of broilers [187,196]. PLF has also integrated many wireless-based sensors and the use of IoT tools to control environmental parameters, with promising results. These sensors can detect and monitor important parameters including temperature, humidity, light intensity, and air quality on a continuous basis. Data are analysed and processed using specific algorithms providing a real-time response on farm’s environmental conditions. Continuous and reliable monitoring requires the use of the internet and advanced technological systems [197,198].

**Table 1:** Summary of technologies used in PLF to monitor different parameters in various animal species. References that use IoT approach are marked with an asterisk (*).

Areas of Interest	Technologies	Cattle	Small Ruminants	Swine	Poultry
Identification and tracking systems	Radio Frequency Identification (RFID)	[45]	[104,105,106,111]	[135,136,139,140]	
	Video frames extraction	[46]		[137,138,141,142]	
	Unmanned Aerial Vehicle (UAV)	[47]	[109,110]		
	Ultra-wideband localization systems (UWB)	[48]	[107]		
	Accelerometers	[49]			
	low-power wide-area network (LPWAN)	[50,51]			
	Video frames extraction		[107]		
	Automatic weighing platform				
Milking systems	AMS robot	[53,54,55,56]	[112,113,114,115]		
	Accelerometers	[53,55]	[118]		
	Electronic gates		[111]		
	Environmental temperature and humidity sensors	[56]			
Oestrus detection systems	Accelerometers	[57,58,59,60]	[116]		
	Ultra-wideband localization systems (UWB)	[61] *			
	Pressure sensors		[117]		
	Infrared thermography (IRT)	[62]	[119,120]		
Diseases detection systems	Automatic weighing platform	[63]			
	Accelerometers	[64,66,67,68,72] *			[180]
	Infrared thermography (IRT)	[65,74]	[121,122,123,124,125]		
	Video frames extraction	[66,67,68,80]	[123]		[175,176,177,178,179]
	Automatic weighing platform	[67]			
	AMS robot	[76,78]			
	Acoustic systems	[79]		[143,145,146,147,148]	[183,184]
	Environmental temperature, air and humidity sensors				[181,182]
	Radio Frequency Identification (RFID)	[80]	[123]		
Animal performances and feed monitoring	Accelerometers	[82,83,84,85,87,88,91,92,93] *	[127]		
	Video frames extraction	[84]		[153,154,155,156,157,158,159] *	[189,196]
	Acoustic systems	[88]			[186,187,188]
	RFID	[94]			
	3D model	[94]			
	Environmental temperature and humidity sensors			[150]	
	Electronic drinkers and feeders			[150,152]	[190] *
Animal behaviour	Accelerometers	[97,98,99,101,102] *	[131]		
	Video frames extraction	[98,99,100]		[170,171,172,173,174]	[177,192,193,194,195] *
	Acoustic systems		[130]	[160,161,162,163,164,165,166,167,168,169]	[189,196]
	Heart beat sensor		[132] *		
	Skin temperature sensor		[132] *	[160,161]	
	Environmental temperature and humidity sensors	[101,102] *			[197,198] *

## 4. Challenges of PLF and IoT

PLF is widely known as an approach for employing inputs efficiently and achieving the greatest possible outputs. Furthermore, PLF is a sustainable system from a variety of perspectives [199]. According to the literature, IoTs will undergo significant technological improvements as service-oriented technology. It is worth noting, however, that the responsibility to leverage the intelligent solutions that IoT will provide in livestock management will remain in human hands. This new monitoring and technology-based approach could pose some challenges in livestock farming systems. The main challenges of this system are summarised below.

### 4.1. Economic Challenges

While the food industry is among the largest in the world, its profit margins are razor-thin and products are usually unstable [200]. The key source of this instability is a direct or indirect reliance on climatic and seasonal influences. 

Every technology function comes at a cost [201]. This type of expense is often unquantifiable, and the dynamics surrounding its estimation are complicated. This all is due to the ever-changing IoT architecture, which stems from a constantly developing framework [202]. Finally, prices rise in remote areas where stable internet service is not always accessible [203,204]. 

Additional challenges for PLF include the continuous development of reliable and efficient sensors, as well as IoT technologies that integrate PLF tools and enable efficient data transmission, storage, security and privacy—all of which requires dedicated professionals with a background in electrical engineering, computer science, biological systems engineering, and other fields, as well as a thorough understanding of farm production systems and livestock biology [27]. As a result, investing in the education of these new animal husbandry professionals is vital.

### 4.2. Applicability

Farmers are rarely experts in life sciences and technology. For these reasons, a specialised service sector is required to maintain technological components, interpret data collected by sensors, formulate and deliver easy, appropriate advice to farmers on a regular basis, and engage users in technological development. Furthermore, it would be desirable to build a fully integrated framework through which all system components could be supplied to end-users in order to promote the functional deployment of PLF systems on farms [205].

A lack of cooperation between researchers, engineers, and technology supplier’s leads to the low adoption rate of PLF technologies on farms. When device components are developed independently from one another, and compete with existing products, PLF production and implementation on farms are prone to failure. Achieving better coordination between PLF tool developers and suppliers represents a significant challenge, but if successful could result in the production of better integrated systems [206]. 

IoT-based systems that are used in PLF commonly involve the use of sensors. It is of importance that such sensors match the reality of farms, without causing an excessive waste of time for farmers, but rather save precious time instead. Otherwise, the advantages of these new systems would be lost. For example, sensors must have long-lasting batteries to allow continuous real-time monitoring to be effective. 

Lastly, PLF systems are often characterized by a certain heterogeneity. Despite considerable efforts to address this problem, further work is needed to establish a balance between the use of reliable, energetically and economically sustainable, intuitive and well-covered systems [207]. 

### 4.3. Ethical Issues

The first potential ethical concern regarding wide implementation of PLF systems is the loss of agricultural jobs and the retraining of remain farmers. As livestock monitoring and care become more automated, fewer hands-on people will be needed on farms, and many of the present skills will become obsolete. 

Agricultural employment and a relationship to food production are often seen as important [208,209], and therefore, losing these jobs could be particularly detrimental for the individual and society. In fact, conventional farming practices allow animals to be treated on an individual basis. In this one-to-one-care culture, there is a characteristic bond favouring the relationship between humans and animals. This classical view of farming would certainly be at risk with the spread of IoT-based systems. In addition, this change could cause farmers to lose interest in the profession and in the relationship with animals [210,211], causing important long-term impacts on the economy and public opinion regarding PLF. Another specific concern originating from this type of technology is the extent to which it is possible for farmers to fulfil their obligations, in order to take care of their animals through this type of closed circuit monitoring technology [34].

Concerns regarding data ownership, how data are used and stored, and who has access to it may also contribute to a lack of faith in technology among farmers and advisors. This can lead to feelings of vulnerability, especially in situations where cameras are constantly recording. Because huge amounts of data are routinely stored in ‘clouds,’ there are also concerns about cyber security [212]. 

Finally, it is critical to reflect on consumer opinion: consumer perceptions must be incorporated into PLF research, as the success or failure of value chains utilizing PLF is ultimately determined by consumer purchasing decisions [213]. Additionally, it has been demonstrated that including customer perspectives early in the process of innovation development enhances social acceptance of technological breakthroughs [214]. As a result, a survey was conducted to determine consumer opinion toward precision livestock production. Despite the numerous benefits discovered during the investigation, the analysis revealed three common consumer concerns: (1) integrating PLF technologies will increase industrialisation in livestock production; (2) PLF technologies and data are vulnerable to misuse and cybercrime; and (3) information about PLF is insufficiently communicated to allow informed purchasing decisions [215].

## 5. Conclusions

The broad use of PLF-based technology has undeniably marked a turning point in the livestock industry. This trend is particularly visible in research, where an increasing number of studies are being conducted regarding the use existing technologies and the development of new and more efficient ones. PLF has become an integral part of many modern farms, allowing farmers to easily monitor animals.

IoT based systems have implemented the efficacy of the PLF approach, thanks to continuous technical advances over the last 20 years and the advent of Industry 4.0. Advanced instruments and sensors are used to monitor animal health and welfare in various ways, hoping to advance agricultural practices worldwide.

Despite excellent results, PLF and Industry 4.0 have their limitations: a part of the public and farmers have brought forward some ethical concerns regarding the fundamental ways that PLF would change the way farms are run. Furthermore, since agricultural profit margins are often very low, the cost of such devices and integrated systems may reduce profitability of farming operations. Finally, the issue of applicability in companies located in rural areas, which often lack sufficient internet connectivity, makes accessing these systems difficult (if not impossible). All of these issues could possibly be overcome in the coming years, as technology improves exponentially each year, allowing people to acquire ever-cheaper, extremely effective devices. Over time, ethically speaking, people tend to embrace change. Globally, the employment of highly technological equipment in daily life has significantly changed people’s lives. The internet is now everywhere in our lives, often without our knowledge. The adoption of these technologies on farms is inevitable and will become as commonplace as smartphone usage is nowadays.

As farms and the livestock industry fully incorporate these technologies, research efforts will have to continue to study the PLF approach, even for the most marginal farming systems, continuously improving the performance of its sensors and operation systems. From this perspective, future objectives may be established: (1) the creation of animal health and welfare standards that are accepted globally, using real-time monitoring technologies; (2) the proper management of so-called “Big Data” in order to effectively simplify an enormous amount of data and to guarantee the privacy of farms while mainly the European Union and other international organizations are investing more and more in these new approaches in order to obtain a high degree of transparency [167]; and (3) the development of environmentally sustainable equipment and sensors, both in terms of construction and disposal. Equipment and sensors must not only be designed for production and health monitoring, but also for the control and improvement of the environmental sustainability of livestock farms [168]; (4) the development of the PLF approach in less frequently reared species, in order to preserve and improve diverse and unique global livestock productions.

In conclusion, the pros and cons of the progressive adoption of PLF and the IoT in the perspective of Agriculture 4.0 are more or less balanced. However, the awareness of an increasingly crowded and globalized world as well as the exponentially increasing demand for animal-based food products could soon be expected to pressure the industry into widespread integration of technologies on farms, fully launching the era of PLF and Agriculture 4.0 once and for all.

## Data Availability

Not applicable.
